# Water-Protein Interactions: The Secret of Protein Dynamics

**DOI:** 10.1155/2013/138916

**Published:** 2013-05-22

**Authors:** Silvia Martini, Claudia Bonechi, Alberto Foletti, Claudio Rossi

**Affiliations:** ^1^Department of Biotechnology, Chemistry and Pharmacy, University of Siena, Via Aldo Moro 2, 53100 Siena, Italy; ^2^Centre for Colloid and Surface Science (CSGI), University of Florence, Via della Lastruccia 3, 50019 Sesto Fiorentino, Italy; ^3^Laboratory of Applied Mathematics and Physics, Department of Innovative Technologies-DTI, University of Applied Sciences of Southern Switzerland (SUPSI), Manno, CH 6928, Switzerland

## Abstract

Water-protein interactions help to maintain flexible conformation conditions which are required for multifunctional protein recognition processes. The intimate relationship between the protein surface and hydration water can be analyzed by studying experimental water properties measured in protein systems in solution. In particular, proteins in solution modify the structure and the dynamics of the bulk water at the solute-solvent interface. The ordering effects of proteins on hydration water are extended for several angstroms. In this paper we propose a method for analyzing the dynamical properties of the water molecules present in the hydration shells of proteins. The approach is based on the analysis of the effects of protein-solvent interactions on water protons NMR relaxation parameters. NMR relaxation parameters, especially the nonselective (*R*_1_^NS^ ) and selective (*R*_1_^SE^ ) spin-lattice relaxation rates of water protons, are useful for investigating the solvent dynamics at the macromolecule-solvent interfaces as well as the perturbation effects caused by the water-macromolecule interactions on the solvent dynamical properties. In this paper we demonstrate that Nuclear Magnetic Resonance Spectroscopy can be used to determine the dynamical contributions of proteins to the water molecules belonging to their hydration shells.

## 1. Introduction

Water-protein interactions play an important role in driving the protein organization at the water interface [1–4]. Water-protein interactions help to maintain flexible conformation conditions which are required for multifunctional protein recognition processes. The intimate relationship between the protein surface and hydration water can be analyzed by studying experimental water properties measured in protein systems in solution. In particular, proteins in solution modify the structure and the dynamics of the bulk water at the solute-solvent interface. The ordering effects of proteins are extended for several angstroms. This process results in a protein hydration shell in which water molecules have restricted dynamics with respect to the bulk water. The extent of interaction can be monitored studying the solvent parameters mostly affected by the presence of a large, slowly reorienting biomacromolecule [5–12]. NMR relaxation parameters, especially the nonselective (*R*_1_^NS^) and selective (*R*_1_^SE^) spin-lattice relaxation rates of water protons, are useful for investigating the solvent dynamics at the macromolecule-solvent interfaces as well as the perturbation effects caused by the water-macromolecule interactions on the solvent dynamical properties [13–25]. In this paper we demonstrate that Nuclear Magnetic Resonance Spectroscopy can be used to determine the dynamical contribution of the biomacromolecules to the water molecules belonging to their hydration shells. In a globular protein solution, three different water environments are present, that is, the buried water molecules (which are integrant part of the protein structure and cannot be removed even during protein crystallization) [3, 4], the water hydration shell around the protein, and the bulk water. The present investigation analyzes the dynamical properties of the water molecules present in the hydration shell around a protein system. Water proton relaxation rates have been used to investigate different systems and phenomena, and theoretical interpretations of the experimental results have been proposed [26–30]. Both the water proton spin-lattice relaxation rates *R*_1_^NS^ and *R*_1_^SE^ in solution are analyzed considering all possible sources of dipolar contributions arising from proton environments. From this analysis an equation for the calculation of ordering effect induced by the macromolecule on the hydration water was derived. In particular the average water rotational correlation time which characterizes water protons dynamics in the protein hydration shell was calculated. This information was then used for the calculation of the dimension of the long range ordering effect caused by the protein molecules on the hydration water.

## 2. Theory

Dipolar nonselective *R*_1_^NS^ and selective *R*_1_^SE^ spin-lattice relaxation rates have the following expressions [31–36]:

(1) $$\begin{array}{c} R_{1}^{\text{NS}}=\sum \rho_{ij}+\sum \sigma_{ij},\\ R_{1}^{\text{SE}}=\sum \rho_{ij}, \end{array}$$

where *ρ*_*ij*_ is the direct self-relaxation rate and *σ*_*ij*_ the “cross-relaxation” rates.

For any *i*, *j* dipolar coupling *R*_1_^NS^ and *R*_1_^SE^ assume the explicit form:

(2) $$\begin{array}{c} R_{1}^{\text{NS}}=\frac{3}{10}\frac{\gamma_{H}^{4}\hbar^{2}}{r_{ij}^{6}}\left[\frac{4\tau_{c}}{1+4\omega_{H}^{2}\tau_{c}^{2}}+\frac{\tau_{c}}{1+\omega_{H}^{2}\tau_{c}^{2}}\right],\\ R_{1}^{\text{SE}}=\frac{1}{10}\frac{\gamma_{H}^{4}\hbar^{2}}{r_{ij}^{6}}\left[\frac{3\tau_{c}}{1+\omega_{H}^{2}\tau_{c}^{2}}+\frac{6\tau_{c}}{1+4\omega_{H}^{2}\tau_{c}^{2}}+\tau_{c}\right], \end{array}$$

where *ħ* is the reduced Plank's constant, *ω*_*H*_ is the proton magnetogyric ratio and Larmor frequency, respectively, *r*_*ij*_ is the internuclear distance, and *τ*_*c*_ is the effective correlation time which modulates the *i*-*j* magnetic interaction.

**Figure 1:**
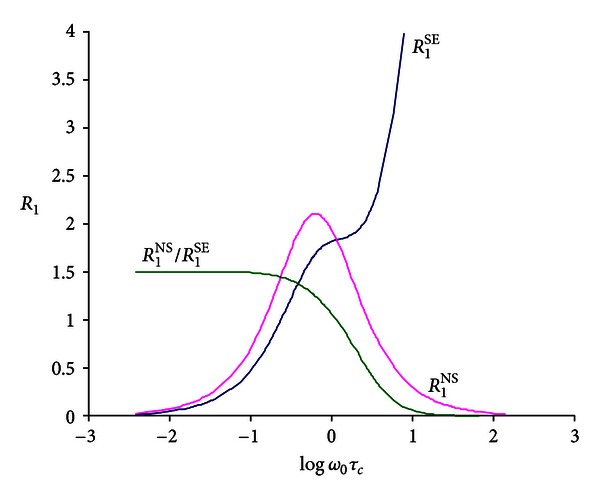
Dependence of selective and nonselective spin-lattice relaxation rates of the motion parameter *ω*_0_ *τ*_*c*_.

The dependence of selective and nonselective spin-lattice relaxation rates of the motion parameter *ω*_0_ *τ*_*c*_ is reported in Figure 1.

In pure water, the water nonselective *wR*_1_^NS^ and selective *wR*_1_^SE^ spin-lattice relaxation rates are

(3) $$\begin{array}{c} wR_{1}^{\text{NS}}=\sum \rho_{ww}^{}+\sum \sigma_{ww}^{},\\ wR_{1}^{\text{SE}}=\sum \rho_{ww}^{}+\sum \sigma_{ww}^{}, \end{array}$$

where *ρ*_*ww*_ and *σ*_*ww*_ are the water direct and cross-relaxation rate contributions which result from water proton-proton intra- and intermolecular interactions. 

In pure water both *wR*_1_^NS^ and *wR*_1_^SE^ assume the same value as the cross-relaxation term *σ*_*ww*_ affects the selective and nonselective measurements equally.

**Figure 2:**
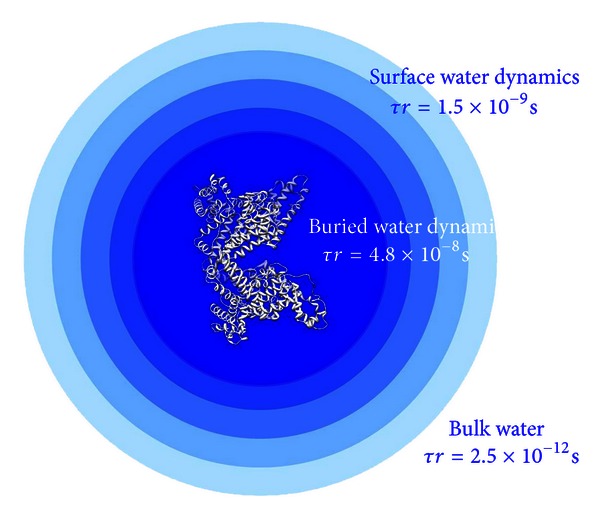
Effect of the ordering effect of proteins on water. Three water environments defined by their dynamical properties can be observed: bulk, surface, and buried water environments.

In binary system (water-protein and/or polymer) we assume the distribution of water molecules as schematically represented by the model showed in Figure 2. Water molecules can be classified into three different categories according to their dynamical properties: (i) bulk water with a typical reorientational correlation time of the order of picoseconds; (ii) water present at the macromolecular surface which exhibits a partially restricted reorientational motion; (iii) buried water molecules. The dynamical properties of the water molecules in these conditions can be well represented by a distribution of correlation time values. These molecules are in fast chemical exchange with the microenvironments present at the protein surface and with the bulk water molecules. These long lived water molecules show dynamics which are mostly determined by the slow reorientation motion of the macromolecule with *τ*_*c*_ values typically of the order of 10^−8^ seconds. These molecules exhibit slow chemical exchange rate in the NMR time scale with the waters present at the macromolecular surface. The contribution of these water molecules to the observed spin-lattice relaxation rates is negligible due to their very low molar fraction.

Relaxometric studies have been used to determine the number and the dominant reorientational correlation time which is involved in the relaxation of water molecules buried in the macromolecular structure [37]. Nevertheless relaxometric experiments cannot monitor the dominant fluctuations which are involved in the relaxation of the water molecules present at the macromolecular surface. In fact this environment is characterized by water molecules which exhibit a distribution of the *τ*_*c*_ values and display fast chemical exchange with other waters of the same environment or with the bulk molecules. These are in fact the appropriate conditions for applying the selective and nonselective water spin-lattice relaxation methodologies.

In water-protein binary systems, under fast chemical exchange conditions between the free (bulk) and bound water, the changes observed in water spin-lattice relaxation rates with respect to the bulk water reflect the presence of water molecules with restricted dynamical reorientation. In these conditions nonselective (*wR*_1_^NS^) and selective (*wR*_1_^SE^) water spin-lattice relaxation rates assume different values as a consequence of a negative protein-water cross-relaxation contribution to *wR*_1_^NS^ and *wR*_1_^SE^. They are defined as

(4) $$\begin{array}{c} wR_{1\exp}=\chi_{b}R_{1b}+\chi_{f}R_{1f}, \end{array}$$

where *wR*_1exp⁡_ is the experimental relaxation rate of water in the presence of the protein, *R*_1*b*_ and *R*_1*f*_ are the water relaxation rates of the pure bound and free environments, and *χ*_*b*_ and *χ*_*f*_ are the molar fraction of water in bound and bulk conditions.

*χ*_*f*_ of the free water molar fraction is assumed to be *χ*_*f*_ = 1 − *χ*_*b*_≅1.

At the bound site, in the presence of D_2_O > 95%, the residual water protons show a relaxation which is mainly dominated by the dipolar interactions with the nonexchangeable protein protons. Water-water interactions (both inter- and intra-) have a sufficient low frequency to be neglected so that

(5) $$\begin{array}{c} wR_{1\exp}^{\text{NS}}=wR_{1}^{\text{NS}}+\chi_{b}\left(\sum \rho_{wp}+\sum \sigma_{wp}\right),\\ wR_{1\exp}^{\text{SE}}=wR_{1}^{\text{SE}}+\chi_{b}\left(\sum \rho_{wp}\right). \end{array}$$

Then the protein contribution to the water relaxation rates, Δ*R*_1_, can be calculated as

(6) $$\begin{array}{c} \Delta R_{1}^{\text{NS}}=wR_{1\exp}^{\text{NS}}-wR_{1}^{\text{NS}}=\chi_{b}\left(\sum \rho_{wp}+\sum \sigma_{wp}\right)=\chi_{b}R_{1b}^{\text{NS}},\\ \Delta R_{1}^{\text{SE}}=wR_{1\exp}^{\text{SE}}-wR_{1}^{\text{SE}}=\chi_{b}\left(\sum \rho_{wp}\right)=\chi_{b}R_{1b}^{\text{SE}}, \end{array}$$

where *R*_1*b*_^NS^ and *R*_1*b*_^SE^ are the relaxation rates of the water molecules present in the bound conditions.

Considering the dependence of the *R*_1_^NS^/*R*_1_^SE^ ratio on *τ*_*c*_ (see (2)), Δ*R*_1_^NS^/Δ*R*_1_^SE^ ratio allows the calculation of the *τ*_*c*_ value resulting from the average contribution of the distribution of motions that characterizes the water dynamics at the macromolecular surface. Equation (2) holds their own validity when a single correlation time value is replaced by a distribution function which considers all different fast exchanging microenvironments. 

In fact

(7) ΔR1NSΔR1SE=χbR1bNSχbR1bSE=R1bNSR1bSE=12τc1/(1+4ωH2τc12)+3τc1/(1+ωH2τc12)6τc1/(1+4ωH2τc12)+3τc1/(1+ωH2τc12)+τc1,

where *τ*_*c*1_ represents a distribution function which considers all individual dynamics which modulate the relaxation. The calculated *τ*_*c*_ value may be not directly related to a physical meaning as the presence at the macromolecular surface of a specific dynamics defined by this value is not demonstrated. Nevertheless this experimentally determined parameter represents the average value which affects the dipolar water-protein interactions at the macromolecular surface. This parameter assumes a value which has to be in between the protein *τ*_*c*_ reorientational motion (~10^−8^ s) and the solvent free tumbling reorientation (~10^−12^ s).

## 3. Materials and Methods

^1^H-NMR spectra were obtained on a Bruker AMX 400 spectrometer operating at 400 MHz. Spin-lattice relaxation rates were measured using the (180°-*τ*-90°-*t*)_*n*_ sequence. The *τ* values used for the selective and nonselective experiments were 0.01, 0.02, 0.04, 0.06, 0.08, 0.1, 0.2, 0.4, 0.8, 1, 1.5, 2, 3, 4, 5, 7, and 10 seconds. The 180° selective inversion of the proton spin population was obtained with a selective perturbation pulse, generated by the decoupler channel. The selective spin-lattice relaxation rates were calculated using the initial slope approximation and subsequent three-parameter exponential regression analysis of the longitudinal recovery curves. The maximum experimental error in the relaxation rate measurements was 5%. 

Human albumin (molecular weight 66200 Dalton) was purchased from Sigma Chemical Co. All the solutions were obtained using D_2_O with a minimum content of deuterium of 99.9%.

## 4. Results and Discussion

The theory presented in the previous section is supported by the experimental results obtained on human albumin system.

Water selective and nonselective spin-lattice relaxation rates as a function of protein concentrations are reported in Table 1. 

**Figure 3:**
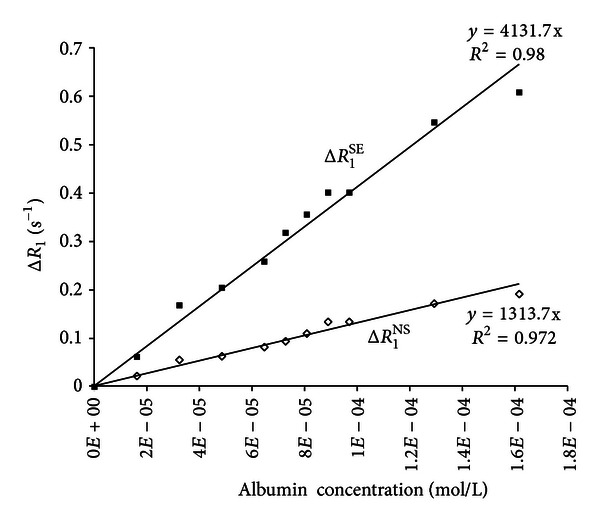
Nonselective and selective proton spin-lattice relaxation rates Δ*R*_1_^SE^ and Δ*R*_1_^NS^ as a function of the human albumin concentration.

The proteins contribution to the water selective Δ*R*_1_^SE^ and nonselective Δ*R*_1_^NS^ relaxation for human albumin systems is shown in Figure 3. In this figure the fitting of the experimental results is also shown. As required by the theory, the calculated straight lines pass through the origin in the system under study. As shown in Figure 3, water selective spin-lattice relaxation rates assume a larger value with respect to the water nonselective spin-lattice relaxation rates, whose results are affected by the negative protein-water cross-relaxation contributions. 

**Figure 4:**
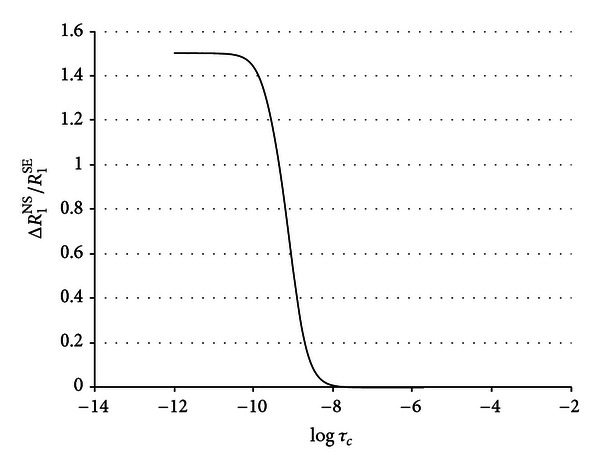
Computed values of Δ*R*_1_^NS^/Δ*R*_1_^SE^ ratio as a function of *τ*_*c*_ at a proton frequency of 400 MHz.

The ratio calculated from the proteins contribution to the water nonselective and selective relaxation rates, Δ*R*_1_^NS^/Δ*R*_1_^SE^, assumes a value of 0.36. The behavior of the Δ*R*_1_^NS^/Δ*R*_1_^SE^ ratio as a function of *τ*_*c*_ is reported in Figure 4. 

**Figure 5:**
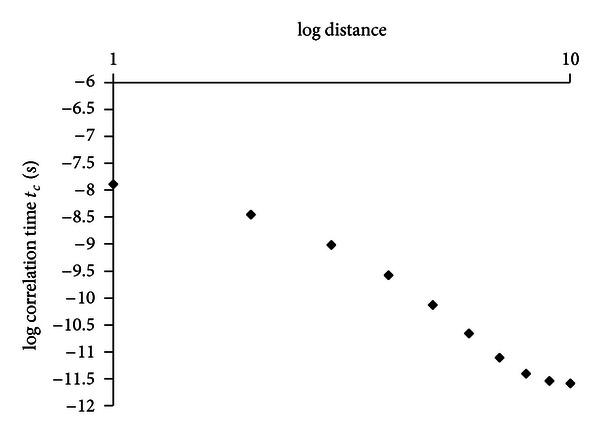
Computed values of the reorientational water correlation times typical of water molecules in the first tenth hydration shells around human albumin. Correlation time was calculated using equation (8) with *a* = 2.5 × 10^−12^, *b* = 4.8 × 10^−8^, and *k* = 1.3. The average correlation time over the ten shells was calculated using the equation *τ*_*c* average_ = ∑_*i*=1_^10^ *χ*_*i*_ *τ*_*ci*_ = 1.5 × 10^−9^ s.

Using the previously computed Δ*R*_1_^NS^/Δ*R*_1_^SE^ ratio of 0.36, an average reorientational correlation time of 1.5 × 10^−9^ s was calculated for the water molecules in the protein hydration shell. In Figure 1 a summary of the water environment typical of protein systems in the case of human albumin is shown: bulk, buried, and hydration water. In the same figure the rotational correlation time values typical of each water environments are reported. The average water hydration correlation time previously computed was used to calculate the ordering effects of the protein on water molecules in the hydration shells at different distance from the protein surface. Assuming a spherical shape with a diameter of 70 Å, the volume of ten hydration spheres around human albumin was calculated. The number of water molecules in each hydration sphere was computed as well as the number of the total water molecules contained in the first ten hydration spheres. Assuming an exponential decay of the water correlation time from its value at the protein surface to the bulk conditions, the following equation was developed:

(8) $$\begin{array}{c} \tau_{c(1,2,\ldots ,10)}=a+be^{-kd}, \end{array}$$

where *τ*_*c*(1,2,…,10)_ are the calculated correlation time values of the water molecules present in the first tenth hydration shell, *a* = 2.5 × 10^−12^ s is the bulk water rotational *τ*_c_, *b* = 4.8 × 10^−8^ s is the buried water rotational *τ*_*c*_,  *d* is the hydration shell distance from the protein surface assumed here to range from 1 to 10 Å, *k* is a constant which defines how strong the ordering effect of the protein on the water molecules. In Figure 4 the computed correlation times (calculated from equation (7)) of the water molecules in each of the first tenth hydration shells as a function of the distance *d* are reported. The convergence between the experimental average reorientational correlation times of the water molecules in the protein hydration shells of 1.5 × 10^−9^ s with the value computed on the basis of (8) was obtained for a *k* equal to 1.3 (Å^−1^). The long range ordering effect of the protein on the hydration water is extended at least to 8 Å (Figure 5).

## 5. Conclusions

In diluted protein solutions, the bulk water proton relaxation shared the contributions from the water molecules in the protein hydration shell. These water molecules differ from the bulk water, mainly because of their correlation times, which is are short for bulk water and longer for the protein hydration waters. In slow motion conditions (*ω*_0_ *τ*_*c*_ ≫ 1, typical of the slow tumbling of protein molecules, these contributions are different: large and positive to *wR*_1_^SE^ and negligible or absent to *wR*_1_^NS^. This process makes *wR*_1_^SE^ larger than *wR*_1_^NS^ as showed in Figure 3. The analysis of both the selective and nonselective water spin-lattice relaxation rates allowed the calculation of the average effective correlation time for the water molecules at the water-protein interface. Moreover, using the assumption of an exponential decay of the rotational correlation time of the hydration water from its value at the protein surface to the bulk conditions, the long range ordering effect of the protein surface on the surrounded water molecules was calculated.

## Figures and Tables

**Table 1:** Water non-selective and selective proton spin-lattice relaxation times as a function of the human albumin content at 298 K. In the same table the protein contribution to the selective and non-selective proton spin-lattice relaxation rates Δ*R*_1_^SE^ and Δ*R*_1_^NS^ is also reported.

Albumin concentration	Albumin concentration	*T*_1_^NS^	*T*_1_^SE^	*R*_1_^NS^	*R*_1_^SE^	Δ*R*_1_^NS^	Δ*R*_1_^SE^
mol/L	mg/mL	s	s	s^−1^	s^−1^	s^−1^	s^−1^
0	0	10.10	10.30	0.099	0.097	0	0
1.6 × 10^−5^	1.0	8.30	6.45	0.120	0.155	0.021	0.058
3.2 × 10^−5^	2.0	7.10	4.55	0.141	0.220	0.042	0.123
4.8 × 10^−5^	3.0	6.15	3.60	0.163	0.278	0.064	0.181
6.5 × 10^−5^	4.0	5.50	2.90	0.182	0.345	0.083	0.248
7.3 × 10^−5^	4.5	5.10	2.70	0.196	0.370	0.097	0.273
8.1 × 10^−5^	5.0	4.70	2.45	0.213	0.408	0.114	0.311
8.9 × 10^−5^	5.5	4.45	2.30	0.225	0.435	0.126	0.338
9.7 × 10^−5^	6.0	4.15	2.10	0.241	0.476	0.142	0.379
1.3 × 10^−4^	8.0	3.55	1.66	0.282	0.602	0.183	0.505
1.6 × 10^−4^	10.0	3.10	1.40	0.323	0.714	0.202	0.559
